# Psychosocial needs of adolescents living with TB in Peru and South Africa

**DOI:** 10.5588/ijtldopen.23.0443

**Published:** 2024-03-01

**Authors:** C. Cintron, G. Hoddinott, B.R. Sinche, R.A. Brown, D.T. Wademan, J.A. Seddon, S.S. Chiang

**Affiliations:** ^1^ Department of Epidemiology, Brown University School of Public Health, Providence, RI, USA;; ^2^ Desmond Tutu TB Centre, Department of Paediatrics and Child Health, Faculty of Medicine and Health Sciences, Stellenbosch University, Cape Town, South Africa;; ^3^ Partners in Health, Lima, Peru;; ^4^ Department of Infectious Disease, Imperial College London, London, UK;; ^5^ Division of Pediatric Infectious Diseases, Department of Pediatrics, Alpert Medical School of Brown University, Providence, RI,; ^6^ Center for International Health Research, Rhode Island Hospital, Providence, RI, USA

**Keywords:** depression, stigma, person-centered care

Dear Editor,

Adolescents (10–19-years-old) account for 11% of the global TB burden,^1^ but they have been largely ignored in TB research and policy. Studies on chronic disease in adolescence have shown that psychosocial factors often negatively impact treatment outcomes.^2^ Nevertheless, there is a paucity of data regarding psychosocial needs of those with TB, especially among adolescents. The WHO’s End TB Strategy promotes person-centered care, which involves evaluating psychosocial factors to enhance the care cascade and improve TB outcomes.^3^ Psychosocial factors (such as stigma, social support and sexual activity) may impact the illness course and engagement in care of adolescents with TB.^4,5^ Research in adults shows that stigma negatively impacts TB care engagement and outcomes, including testing for HIV.^6^ TB-related stigma may also affect an adolescents’ general well-being by inhibiting school attendance or social engagement, leading to increased social isolation.^7^ In this study, we examined the psychosocial profile of adolescents with TB to inform person-centered care of this population.

We analyzed data from two prospective cohort studies of adolescents on TB treatment, one in Lima, Peru, and the other in Cape Town, South Africa. Adolescents were recruited between 2020 and 2022 at local health centers: in Lima, adolescents with rifampicin-susceptible TB of any anatomic site who were within the first 5 weeks of treatment were eligible for the study; in Cape Town, adolescents were eligible if they had newly diagnosed pulmonary TB with any drug susceptibility pattern and were within 14 days of treatment initiation. Comprehensive psychosocial surveys, which included scales that were previously validated (PHQ-9 for depression,^8^ AUDIT for alcohol use,^9^ an adapted Van Rie Scale for TB-related stigma^10^) or newly developed (caregiver support, TB knowledge) were conducted between Weeks 3 and 5 of therapy in Peru and after 8 weeks of therapy in South Africa. The surveys were self-administered in Peru and interviewer-administered in South Africa. Cronbach’s α was used to assess internal consistency of the newly created scales. We considered α ≥0.8 to indicate good consistency. We compared variables between the two cohorts using the χ^2^ and Wilcoxon rank-sum tests. Written informed consent was obtained from participants aged ≥18 years, and consent was sought from the parents or legal guardians for adolescents <18 years, with written assent from the participant. This research was approved by the institutional review boards of the National Institute of Health of Peru, Lima, Peru; Rhode Island Hospital, Providence, RI, USA; and Stellenbosch University, Cape Town, South Africa.

The analysis consisted of 297 adolescents, of whom 249 were in Peru and 48 in South Africa (Table). In Peru, 26 (10.4%) had extrapulmonary TB, whereas 3 (1.2%) had both pulmonary and extrapulmonary TB. In South Africa, 10 (20.8%) had multidrug-resistant TB (MDR-TB). One participant (0.4%) in Peru and 4 (8%) in South Africa were living with HIV. There were no differences in age (median: 17 years) between the cohorts. Males represented 63.9% of the sample in Peru, compared to 39.6% in South Africa. Overall, 38.7% of adolescents reported being sexually active, although this finding was driven mainly by 18–19-year olds (51% sexually active). Adolescents at both sites had high scores on the depression screening tool, with most reporting moderate or severe scores. In both countries, but especially in South Africa, many participants reported experiencing TB-related stigma. For example, 78% of participants expressed fears of TB diagnosis disclosure. Low levels of family support and TB knowledge were reported across sites. In South Africa, there was evidence of problematic alcohol use. Finally, although over half of participants reported no missed meals due to lack of money, more adolescents in South Africa missed meals than in Peru. Tobacco use was higher among males than females (23.6% vs. 12.0%; *P* = 0.026). Additionally, males had higher caregiver support and TB knowledge scores, although there was little difference in effect size (Supplementary Table S1).

**Table.** Demographic and psychosocial characteristics.

	Overall	Peru	South Africa	*P*-value
	(*n* = 297)	(*n* = 249)	(*n* = 48)	
	*n* (%)	*n* (%)	*n* (%)	
Male sex	178 (59.9)	159 (63.9)	19 (39.6)	0.003
Age, years, median [IQR]	17 [15‒18]	17 [15‒18]	17 [15‒18]	0.379
Stigma score,* median [IQR]	23 [18‒30]	22 [17‒26]	29 [21‒33.3]	<0.001
Depression screening score PHQ-9,^†^ median [IQR]	10 [6‒14]	9 [5‒13]	13 [11‒17]	<0.001
AUDIT score, median (range) [IQR]	0 (0–26) [0‒0]	0 (0–26) [0‒0]	3.50 (0–26) [0‒8.3]	<0.001
Sexually active	106 (38.7)	86 (38.1)	20 (41.7)	0.761
Caregiver support score,^‡^ median [IQR]	18 [13.5‒20]	19 [16‒20]	6 [4‒6]	<0.001
TB knowledge score,^§^ median [IQR]	11 [9‒12]	12 [10‒12]	6 [5.8‒6]	<0.001
Missed meals due to poverty				<0.001
Never (0 days/week)	150 (51.7)	127 (52.5)	23 (47.9)	
Rarely (1–2 days/week)	65 (22.4)	63 (26.0)	2 ( 4.2)	
Sometimes (3–4 days/week)	36 (12.4)	22 ( 9.1)	14 (29.2)	
Often (5–6 days/week)	14 (4.8)	7 ( 2.9)	7 (14.6)	
Always (7 days/week)	25 (8.6)	23 ( 9.5)	2 ( 4.2)	
Illicit drug use in the last 12 months	55 (18.5)	35 (14.1)	20 (41.7)	<0.001

* Stigma score range 10–50; scale assessed for internal reliability in this population (Cronbach’s alpha = 0.76).

^†^ PHQ-9 (depression screening) score range 0–27: 0–4, minimal depression; 5–9, mild depression; 10–14, moderate depression; ≥15, severe depression.

^‡^ Caregiver support scale, 4 items, score range 5–25; scale assessed for internal reliability in this population (Cronbach’s alpha = 0.95).

^§^ TB knowledge scale, 3 items, score range 3–12; scale assessed for internal reliability in this population (Cronbach’s alpha = 0.88).

IQR = interquartile range; PHQ-9 = Patient Health Questionnaire-9; AUDIT = Alcohol Use Disorders Identification Test.

These findings demonstrate that adolescents with TB in two different settings experienced frequent stigma, depression and low levels of caregiver support. This highlights the need for interventions to improve mental health and support for patients, as these factors not only impact the TB illness trajectory but also patients’ overall wellbeing.^7,11,12^ The wide range of unmet needs among the participants may be addressed through age-appropriate care models. HIV research has shown positive outcomes such as increased testing, linkage to care, and treatment retention associated with differentiated adolescent care models, including decentralized treatment access, adolescent clinic hours, and peer and community support clubs.^13,14^ Providing designated time and space for adolescent treatment would reduce TB-related stigma experienced at clinics and may reduce symptoms of depression, which often stem from stigmatizing care experiences.^13,14^ Peer and community support outlets would provide and/or improve social networks and may further alleviate stigma and depression sequelae. A differentiated care model for adolescents with TB may also be linked with other key adolescent healthcare services, such as screening for sexually transmitted infections (STIs) to increase care engagement and overall wellbeing.^13^

The observed differences in survey responses between sites underscores the need for setting-specific evaluations of the psychosocial needs of this vulnerable population. Although our work found important associations, it is not without limitations. These populations differed in a wide range of social, psychological and economic factors, which may have impacted their feelings towards and experiences with TB. Furthermore, societal elements and health services are likely to be quite different in their relationship to TB depending on geographic location. In those diagnosed with TB, there is a substantial difference in HIV prevalence between Peru (5.9%) and South Africa (57%).^15^ Co-infection with HIV likely impacts and exacerbates adolescents’ experience with TB-related mental health, stigma and interpersonal relationships; however, as five adolescents in our sample were living with HIV, the study was underpowered to explore these potential differences. In South Africa, TB is still associated with HIV and many with TB experience proxy HIV stigma. Furthermore, in South Africa, there were more participants with MDR-TB, which may have impacted our results, and more research is needed to explore the specific psychosocial needs of adolescents with MDR-TB.

In this analysis, we identified multiple psychosocial needs among adolescents with TB. Implementing person-centered care – a cornerstone of the End TB Strategy – will optimize health outcomes among adolescents.

## Supplementary Material


