# Supplementary material for: Psychosocial needs of adolescents living with TB in Peru and South Africa
Source: IJTLD Open. 2024 Mar 1;1(3):147–9. doi: 10.5588/ijtldopen.23.0443 (PMC11221585; doi:10.5588/ijtldopen.23.0443)
Supplement: Supplementary file 1 [file iutld_ijtld_open_23.0443_supplementarydata1.pdf]

©2024 The Union http://dx.doi.org/10.5588/ijtldopen.23.0443

Psychosocial needs of adolescents living with TB in Peru and South Africa

Supplementary Table S1. Psychosocial characteristics by participant gender.

	Female (n=119)	Male (n=178)	p-value
**Age (mean (SD))**	16.6 (2.3)	16.6 (2.1)	0.9
**Missed meals due to poverty, n (%)**			0.246
**Never (0 days/week)**	66 (55.9)	84 (48.8)	
**Rarely (1-2 days/week)**	20 (16.9)	45 (26.2)	
**Sometimes (3-4 days/week)**	18 (15.3)	18 (10.5)	
**Often (5-6 days/week)**	6 (5.1)	8 (4.7)	
**Always (7 days/week)**	8 (6.8)	17 (9.9)	
**Stigma score (mean (SD))**	23.8 (8.2)	24.2 (8.4)	0.693
**PHQ-9 (mean (SD))**	11.0 (5.6)	10.0 (5.6)	0.137
**AUDIT score (mean (SD))**	1.2 (2.9)	1.5 (4.4)	0.481
**Sexually active, n (%)**	41 (36.0)	65 (40.6)	0.513
**Caregiver support score (mean (SD))**	14.6 (5.9)	16.3 (4.8)	0.008
**TB knowledge score (mean (SD))**	9.7 (2.6)	10.5 (2.1)	0.002
**Tobacco use, n (%)**	12 (10.1)	41 (23.6)	0.026
**Illicit drug use within last 12 months, n (%)**	17 (14.3)	38 (21.3)	0.167

[§] Caregiver support scale, 4 items, score range 5-25; scale assessed for internal reliability in this population (Cronbach's alpha = 0.95)

[§§] TB knowledge scale, 3 items, score range 3-12; scale assessed for internal reliability in this population (Cronbach's alpha = 0.88)
